# MolCompass: multi-tool for the navigation in chemical space and visual validation of QSAR/QSPR models

**DOI:** 10.1186/s13321-024-00888-z

**Published:** 2024-08-12

**Authors:** Sergey Sosnin

**Affiliations:** Department of Pharmaceutical Sciences, Faculty of Life Sciences, University of Vienna, Josef-Holaubek-Platz 2, 1090 Vienna, Austria

**Keywords:** Chemical space visualization, Clustering, Applicability domain, Visual validation, Chemoinformatics, QSAR/QSPR modelling

## Abstract

The exponential growth of data is challenging for humans because their ability to analyze data is limited. Especially in chemistry, there is a demand for tools that can visualize molecular datasets in a convenient graphical way. We propose a new, ready-to-use, multi-tool, and open-source framework for visualizing and navigating chemical space. This framework adheres to the low-code/no-code (LCNC) paradigm, providing a KNIME node, a web-based tool, and a Python package, making it accessible to a broad cheminformatics community. The core technique of the MolCompass framework employs a pre-trained parametric t-SNE model. We demonstrate how this framework can be adapted for the visualisation of chemical space and visual validation of binary classification QSAR/QSPR models, revealing their weaknesses and identifying model cliffs. All parts of the framework are publicly available on GitHub, providing accessibility to the broad scientific community.

**Scientific contribution**

We provide an open-source, ready-to-use set of tools for the visualization of chemical space. These tools can be insightful for chemists to analyze compound datasets and for the visual validation of QSAR/QSPR models.

## Introduction

Nowadays, there is a significant increase in the generation and storage of chemical data in the field of medicinal and organic chemistry. This process can be described as the sunrise of the “Big Data” era in chemistry [1]. However, this increase in data poses a challenge, limiting humans’ abilities to analyze and process such large datasets effectively. Development of tools that represent chemical data in a handy graphical way can augment human’s abilities to analyze large datasets.

The key component in chemical data processing are typically molecules. The general idea of interactive chemical visualization is to represent molecules on a scatter plot, preserving their chemical similarity, while at the same time visualizing additional information using color or size of the points. However, automated preprocessing of chemical data usually necessitates programming skills. A possible option is taking advantage of low-code/no-code (LCNC) solutions, for example, KNIME [2] or Pipeline Pilot [3] where the program logic can be described as a programmable flowchart.

Recently, several studies on the application of parametric t-SNE models for analyzing chemical data [4–6] have been published. However, the use of parametric t-SNE, even for applying an already trained model, requires advanced programming skills. To broaden the scope of potential users in general, and to address a specific problem of the visual validation of QSAR/QSPR models in particular, we have created a set of easy-to-use tools for the visual analysis of chemical space. Aiming to strike a balance between ease of use and flexibility, we have implemented it across three distinct tools. The first is a Python library, designed to offer maximum flexibility for programmers. Next, we introduced a KNIME node, which seamlessly integrates into LCNC KNIME pipelines. Lastly, a GUI tool has been developed, ready for immediate use, facilitating straightforward visualizations right out of the box with the focus on the visual validation of QSAR/QSPR models.

The discussion about chemical data visualization can be fundamentally segmented into three essential subtopics: (i) computational approaches focused on dimensionality reduction, (ii) technical implementation involving visualization engines and corresponding tools, and (iii) the objectives driving the visualization process. Each of these elements holds substantial relevance to the study, and as such, we plan to underscore and elaborate on each of these subtopics below.

### Computational approaches for the chemical data visualization

In cheminformatics, chemical compounds are typically represented as molecular graphs. However, in many cases, graph representation is not convenient for numerical processing. Instead, numerical descriptions, in the form of highly-dimensional vectors that reflect essential properties such as structural, physico-chemical, and quantum characteristics of molecules, are utilized. Humans, however, cannot mentally operate in highly-dimensional spaces. Thus, from a mathematical viewpoint, the visualization problem essentially becomes a dimensionality reduction problem. Recently, several methods and tools have been proposed for the visualization of chemical space [7, 8]. One of the first methods proposed for dimensionality reduction was Principal Component Analysis (PCA) [9]. This method performs a linear transformation of the data into a number of principal components. This method is fast and well-studied; however, the linear nature of projections restricts the ability to handle complex structures of the original high-dimensional space. That, in practice, can lead to low discrimination ability and crowding problems [10]. The Self-Organized maps (SOM) method was proposed as a non-linear approach for data visualization and virtual screening [11]. Further, Generative topological mapping (GTM) was proposed for the visualization and analysis of chemical space space [12]. Multidimensional scaling (MDS) is also widely used to visualize chemical data. [13].

T-distributed Stochastic Neighbor Embedding (t-SNE) is one of the most popular methods for dimensionality reduction [14]. Proposed in 2008, this method rapidly gained popularity for visualizing high-dimensional data in various areas of science and technology, including chemistry [15]. t-SNE minimizes the Kullback-Leibler divergence between high- and low-dimensional statistical distributions, in the way to keep the pairwise distances between data points in the high-dimensional space when mapped to a lower-dimensional space. Nevertheless, the t-SNE method comes with several limitations. Firstly, it demands substantial computational resources, particularly when dealing with extensive high-dimensional datasets. Secondly, the method is inherently non-deterministic; due to its stochastic nature and random initialization, it yields different embeddings upon different executions, preventing the preservation of a consistent global chemical map when applied to new compounds. Numerous strategies have been introduced to address these challenges. A notable advancement was made by Probst et al., the team proposed the Tree MAP (TMAP) method, a novel approach that mitigates these issues [16]. This model is based on a graph theory and represents data in the form of an extensive tree structure. The author demonstrated that TMAP can process datasets with more than $$ 10^7$$ compounds, keeping local features as soon as the global chemical space structure of the original high-dimensional space. Another possible approach, named parametric t-SNE, was proposed [17] by the author of the original t-SNE. Then, this method was adopted for the visualization of chemical compounds [4] and chemical reactions [5].

The parametric t-SNE method employs an artificial neural network as its core mechanism, projecting chemical structures onto a 2D plane. The model is parameterized by the neural network weights, and it is trained to group structurally similar compounds together, leading to the formation of scaffold-based clusters. This approach possesses a deterministic nature, which distinguishes it from traditional t-SNE.

Once trained, the neural network in the parametric t-SNE method can consistently project new compounds into predefined regions of the 2D space. This deterministic characteristic opens new possibilities for the automated description and exploration of chemical spaces. It enables researchers to refer to specific regions of the chemical space in a manner like geographical coordinates, which open doors for many interenting applications, one of this has been demonstrated in the author’s PhD thesis [18], where it was shown that chemical compounds with desired properties could be sampled from specific regions of the chemical space using generative artificial neural networks.

It should be stressed that we consider determinism solely in the context of applying the model to new compounds. Since the parameters of the neural networks remain constant after training, the projection of new compounds will also be deterministic. This means that if a pre-trained parametric t-SNE model projects a specific scaffold into a fixed-coordinate region on a 2D map, the projections of new compounds with the same scaffold will fall into the same region with fixed coordinates. At the same time, the training is not deterministic, should someone retrain the model, the cluster with the corresponding scaffold would still be visible, though it may migrate to any region of the new map. However, in this paper, we do not discuss the retraining of parametric t-SNE models, and under this assumption, parametric t-SNE modeling can be considered deterministic.

### Tools for the visualisation of structural data

Over the years, numerous community efforts have been dedicated to the development of chemical data visualization software. Some of these tools exist as standalone programs or web tools, while others are integrated as plugins into popular data analysis platforms such as KNIME [2] and Cytoscape [19]. These plugins and tools typically employ dimensionality reduction techniques and graph-based algorithms, as previously described, to cluster compounds effectively. For instance, Scaffold Hunter is a free software that specializes in the visualization of chemical data, utilizing scaffold clustering to visualize chemical space and represent it through various views such as dendrograms, heat maps, and clouds [20]. Another example includes HiTSEE KNIME [21], an extension for the KNIME platform designed for analyzing large chemical screens and navigating chemical spaces. Additional notable chemical visualization tools include ChemGPS-NP [22], SAR-maps [23], and SARANEA [24].

### Visual validation of QSAR/QSPR models

QSAR/QSPR models have been used for decades to predict the properties of organic compounds computationally, eliminating the need for expensive real-world experiments. These methods have become particularly important because they pave the way for a fully non-animal risk assessment of chemicals [25]. However, over time, these models have become increasingly sophisticated, evolving into what is often referred to as “black-box” modeling. Such complexity is undesirable, particularly when these models are intended to be used for regulatory purposes. The limited understanding of the Applicability Domain (AD) of a model, which affects their overall trustworthiness.

There are several numerical methods available to estimate a model’s Applicability Domain, but these often fall to provide insights necessary for interpreting the model’s performance across various regions of chemical space. This work aims to address this limitation.

The concept of visual validation of QSAR/QSPR models has been recently proposed [26]. This approach allows for the visualization of a model’s chemical space, using, for instance, compounds from a validation set, and employs color or size encoding to represent predictions and errors. Such a representation makes it easier to identify compounds (or regions of chemical space) where the model’s predictions are not satisfactory, enabling a more systematic analysis and refinement of the model. This idea has been implemented in the CheS-Mapper 2.0 tool [26], demonstrating its applicability across various datasets.

## Implementation

**Fig. 1:**
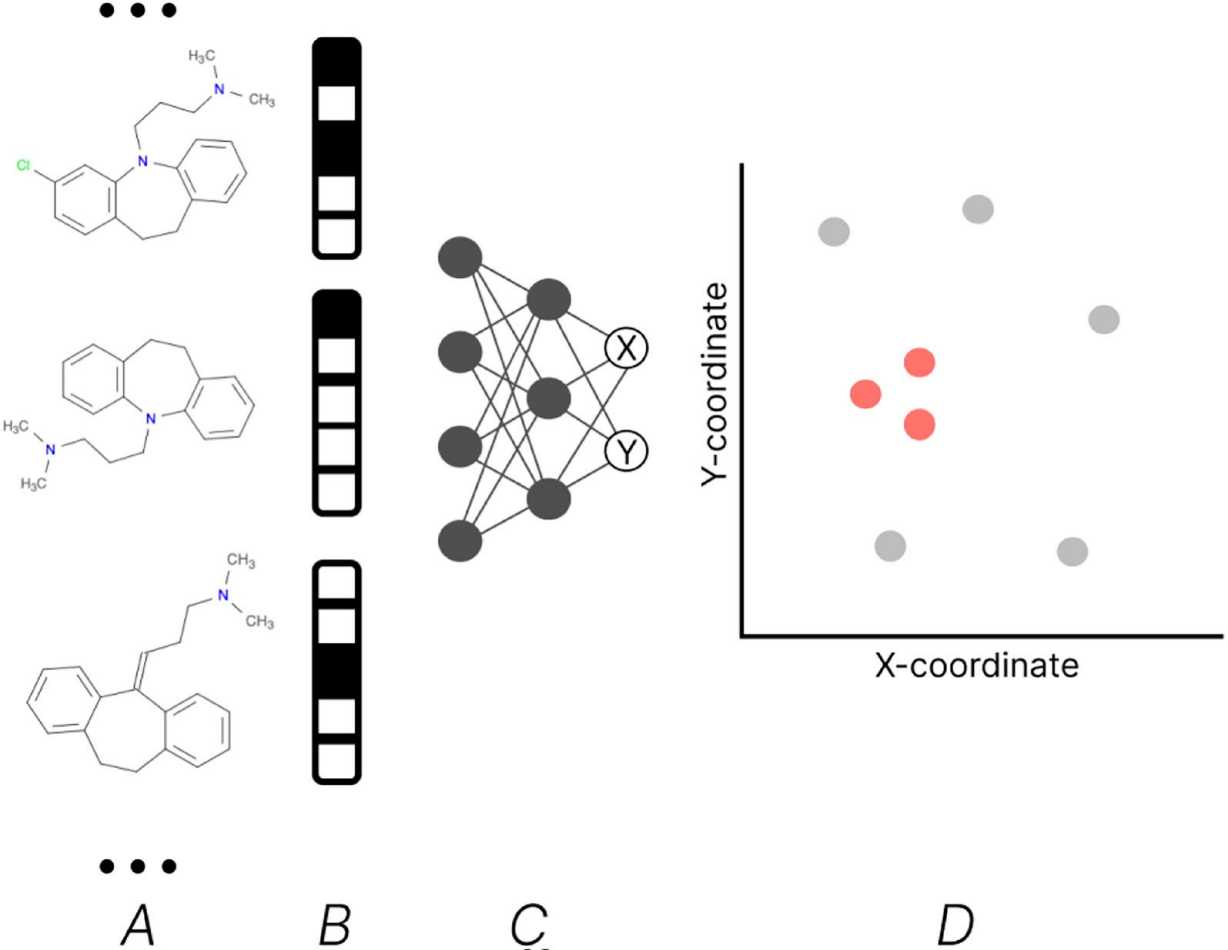
Application of a Parametric t-SNE model: A set of chemical compounds (**A**) is converted into ECFP binary fingerprints of a fixed length (**B**). Then, a pretrained artificial neural network (ANN) (**C**) projects these fingerprints into coordinates, forming 2D clusters where structurally similar compounds are grouped together (**D**)

The core technique in MolCompass is parametric t-SNE. The primary idea of this method is to project chemical compounds onto a 2D plane while preserving their chemical similarity. The original (non-parametric) t-SNE method models points in a high-dimensional space (the space of chemical descriptors) and a low-dimensional space (the 2D plane onto which the chemical compounds are projected) as statistical distributions. The algorithm then minimizes the Kullback-Leibler (KL) divergence between these distributions by optimizing the coordinates of points in the low-dimensional space. The parametric t-SNE version uses a feed-forward artificial neural network (ANN) as a projector from a high-dimensional space to a low-dimensional space. The inputs to the ANN are fed by chemical descriptors , and it produces two outputs that correspond to the coordinates X and Y on the 2D plane. This neural network was trained using KL-divergence as the loss function, effectively learning the projection. The scheme of the application of the trained model is illustrated in Fig. 1. The model was trained using molecular structures from ChEMBL v.23. The dataset contained 1,564,049 molecular structures. These SMILES representations were standardized with the molvs Python package, and then used to compute ECFP fingerprints (2048-bit length binary ECFP fingerprints with radius=3). Jaccard distance was used as the distance in the high-dimensional space. The full technical details regarding the training and performance of the original parametric t-SNE model can be found in the article [4].

MolCompass comprises three components. The first is a Python library, *molcomplib*, which is the computational core for projecting chemical compounds. It includes a pre-trained parametric t-SNE model that facilitates the programmatic processing of structural data. Additionally, we prepared a KNIME extension (node) equipped with this parametric t-SNE model. Finally, we provide a GUI tool (*MolCompassViewer*) that implements the techniques for visual validation and analysis of QSAR/QSPR models. In the subsections below, we will describe these three parts individually.

### Molcomplib python package

We prodive a user-friendly, lightweight package containing the MolCompass model, designed for easy maintenance and installation. It can be seamlessly installed using the standard *pip* installer. The package has minimal dependencies, requiring only *numpy* and *rdkit*.

**Fig. 2:**
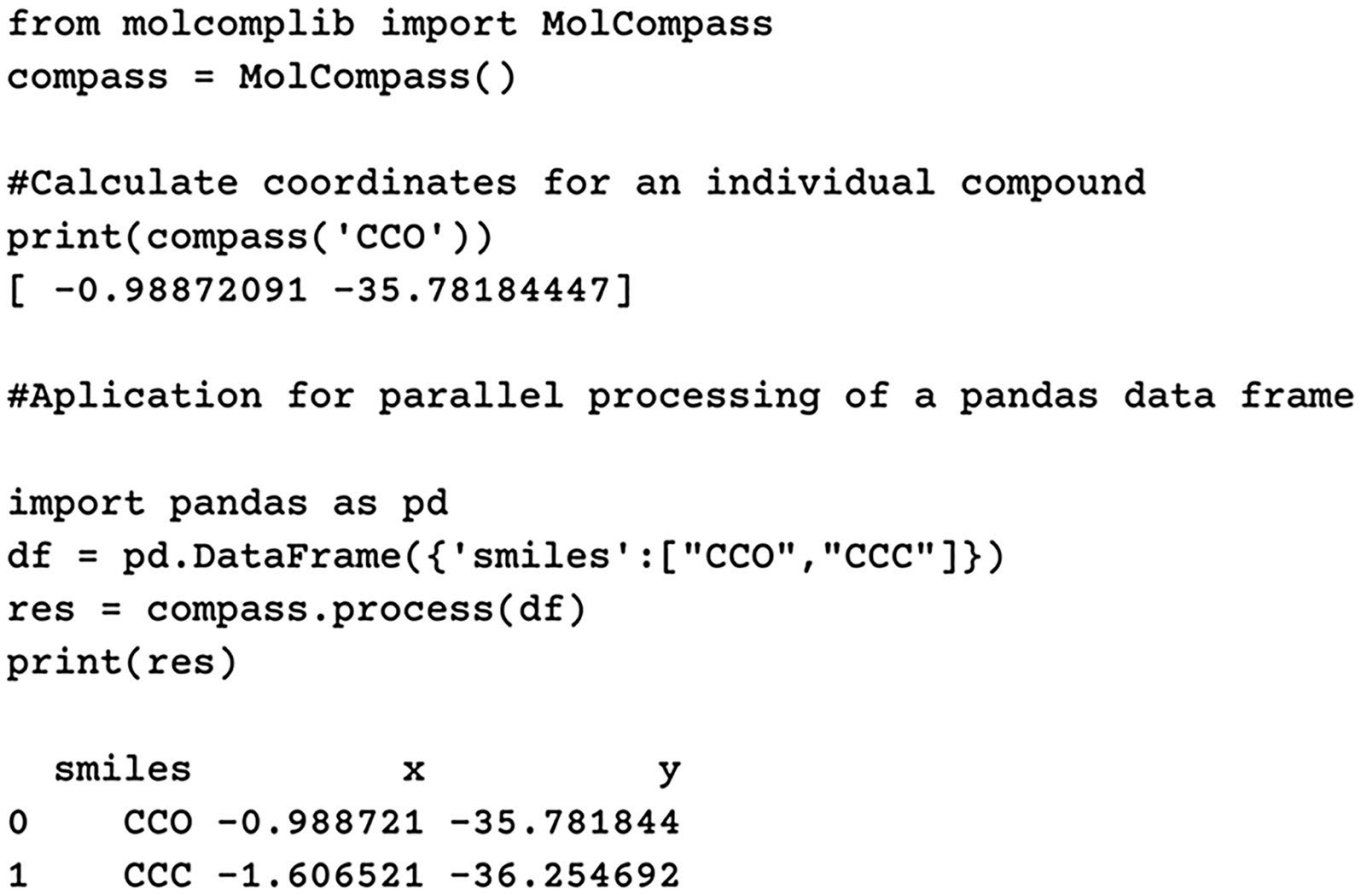
*Molcomplib* library demo example

An option for parallel processing is available, enabling users to leverage the *pandas* interface with the *pandarallel* package for enhanced performance. The usage of this package is straightforward, involving simple steps such as importing the MolCompass object and applying it to molecules, represented either as SMILES strings or within pandas data frames for parallel processing. When bound to pandas, MolCompass efficiently scales across multiple processor cores.

Originally, the parametric t-SNE model [4] was trained using PyTorch. However, we have since transitioned the model to rely on NumPy arrays, eliminating the dependence on PyTorch. Consequently, the final version of *molcomplib* primarily depends on rdkit and NumPy. This strategic simplification prevents the onset of “dependency hell”–a challenging scenario where a plethora of dependencies either complicates resolution or necessitates the installation of an excessive number of additional packages. A basic usage example of *molcomplib* is illustrated in Fig. 2.

#### KNIME node

The Konstanz Information Miner (KNIME) is a visual programming platform for data analysis. Originally developed for bioscience applications, it has maintained popularity in both chemo- and bioinformatics sectors [2]. KNIME’s philosophy revolves around a data flow that navigates through specific processing nodes, where each node is designed to perform a singular operation on the data. KNIME is written in the Java programming language. However, starting from version 4.6, KNIME introduced a native Python interface.

To enhance KNIME’s functionality, we developed a MolCompass KNIME node, leveraging the native Python interface available from version 4.6 onwards. This node accepts a KNIME data table containing molecules represented as SMILES strings and outputs the table with computed coordinates. Such coordinates can be paired with other KNIME nodes for the visualization and exploration of chemical space.

**Fig. 3:**
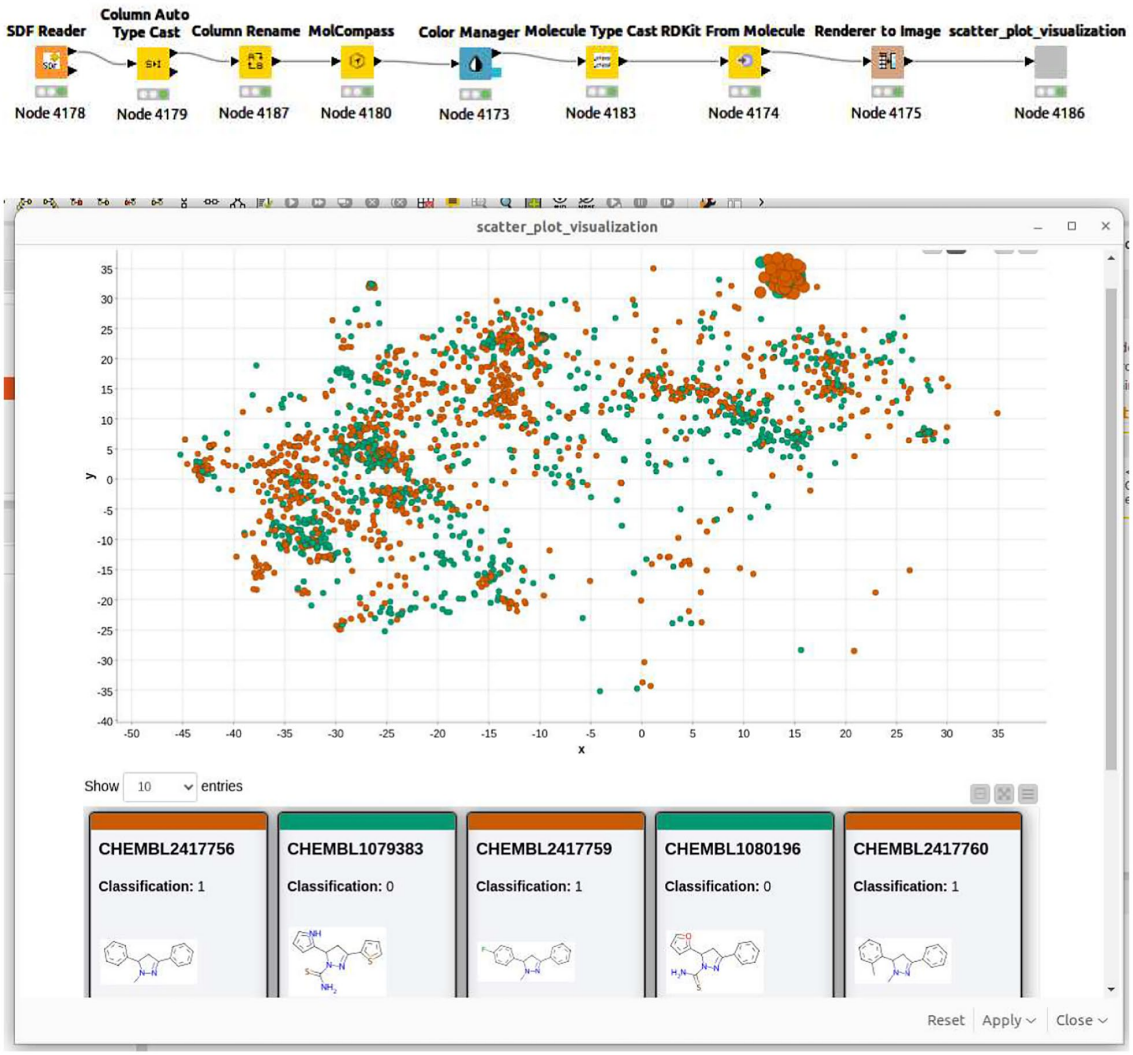
The example of an advanced KNIME workflow (top). This workflow creates a KNIME tool for chemical space analysis and chemical data management. A screenshot of the KNIME visualization of chemical space resulted from the workflow (bottom)

Figure 3 showcases an example of how this extension can be utilized for chemical space analysis. This workflow delivers an interactive scatter plot, color-coded based on properties chosen by the user. It offers standard navigation, including zoom and selection. Moreover, users can view the structural composition of selected compounds, along with their respective activity types. The MolCompass KNIME workflow is designed for flexibility, allowing extensive customization based on user requirements.

#### MolCompass application (MolCompassViewer)

**Fig. 4:**
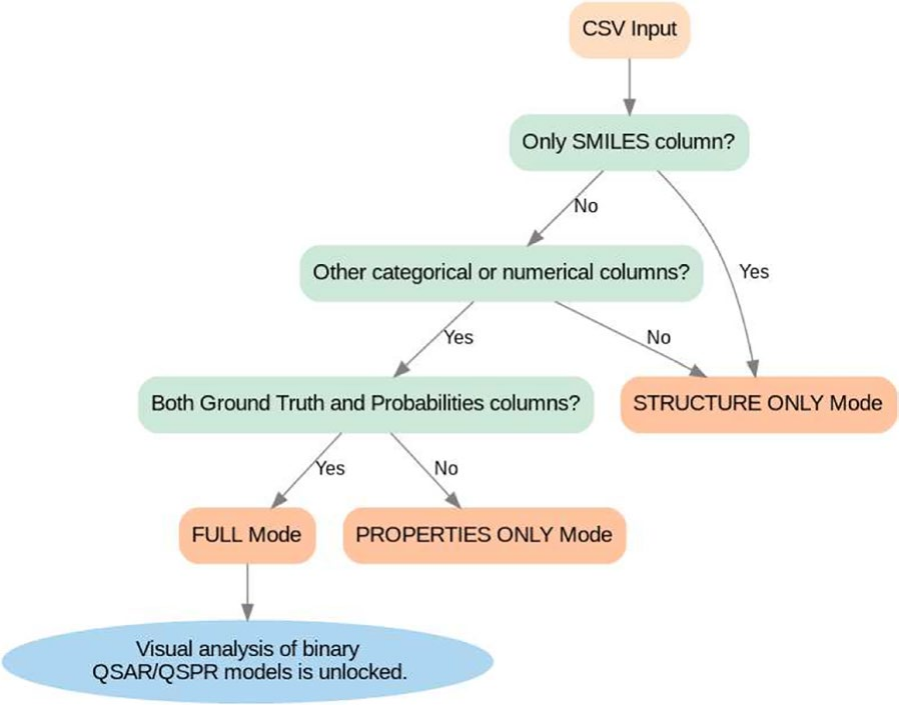
A flowchart demonstrating the process of mode selection based on the structure of the input CSV file

This tool facilitates the visualization of chemical space, applicability domain analysis, and visual validation of QSAR/QSPR models. Developed in Python, it utilizes the *plotly* and *dash* data visualization libraries. Though the primary aim of *MolCompassViewer* is the visual analysis for QSAR/QSPR models, it is flexible, allowing for visualization based just on structures with linked information or even structures alone.

**Fig. 5:**
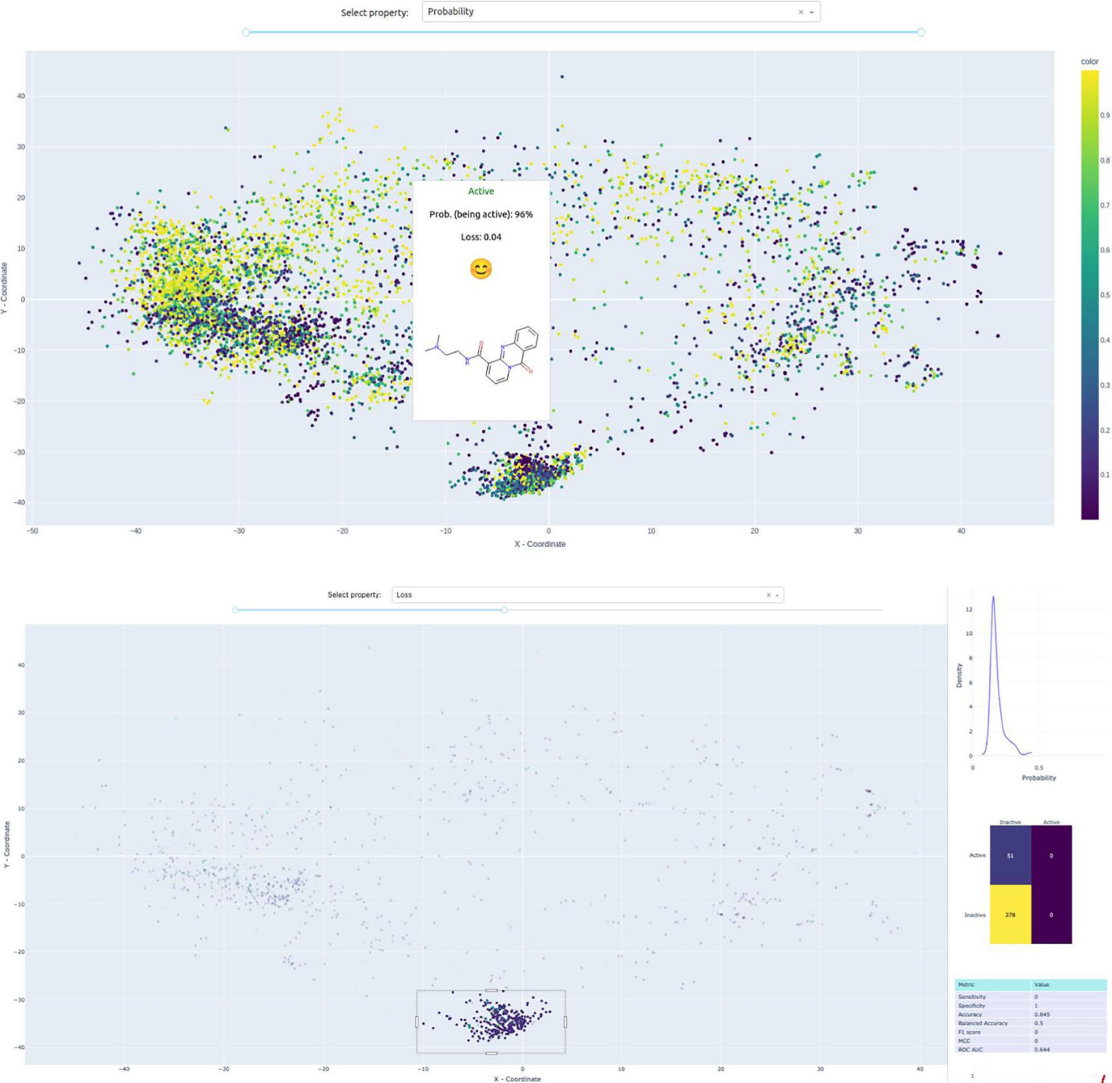
A screenshot demonstrating the interface of MolCompassViewer in FULL mode for individual compounds analysis (top). The demonstration of statistical parameters of a binary QSAR/QSPR model for the selected region of interest (bottom). The visualised data is: Estrogen Binders dataset (see sec. Reference model)

Users simply need to provide the path to a CSV file for visualization. MolCompassViewer uses heuristics to determine the types of columns within the CSV file and chooses an operational mode accordingly. The basic requirement is the presence of a column with molecular structures encoded as SMILES strings.

- *STRUCTURE ONLY*: If only the SMILES column is found, the tool operates in this mode. In this mode, additional visualizations, such as color layers and analysis of QSAR/QSPR models, are unavailable.
- *PROPERTIES ONLY*: If additional columns, either categorical or numerical, are present alongside the SMILES column but it doesn’t have both Ground Truth and Probabilities columns, the viewer switches to this mode. This mode prioritizes the analysis of chemical space, with a focus on examining compounds’ datasets rather than the models. Here, each point in the visualization is colored based on user-selected properties.
- *FULL*: This mode is exclusively for a comprehensive visual analysis of binary QSAR/QSPR models. It is activated if the CSV file includes SMILES strings, Ground Truth, and predicted probabilities columns. Special features, such as visual analysis of the binary QSAR/QSPR models, become accessible in this mode.

This process is demonstrated in Fig. 4.

In Fig. 5 (top), we present a screenshot of the *MolCompassViewer* app. The central feature is a scatter plot representing the chemical space under study, where each point corresponds to a molecule. When a user hovers over a point, a pop-up displays the molecular structure and related information. In FULL mode, users can color the plot based on at least three parameters, depending on the dataset: Ground Truth, Probabilities, and Loss. If the CSV file contains additional categorical or numerical columns, they can also be selected for visualization. Users can choose the desired property using the dropdown menu at the top. Additionally, the interface offers standard scatter plot features, such as zooming and panning. The default color space adjusts to the minimum and maximum range of the property of interest across all data points. However, some molecules with exceptionally high values might skew the coloring. To address this, a range slider is available below the dropdown menu to fine-tune the color range.

Visual analysis of binary QSAR/QSPR models necessitates the inclusion of molecular structures, probabilities indicating whether compounds are active (ranging from 0 for inactive to 1 for active), and ground truth values. This analysis is exclusively available in FULL mode. To initiate this, users should select an area within the chemical space using the picker tool. For all compounds in the selected area, binary classification statistical parameters are calculated:

- sensitivity
- specificity
- accuracy
- balanced accuracy
- F1 score
- Matthews correlation coefficient
- AUC ROC

Additionally, charts displaying the KDE approximation of probability distributions, the ROC curve, and confusion matrices can be viewed (see Fig. 5 bottom).

## Results and discussion

**Fig. 6:**
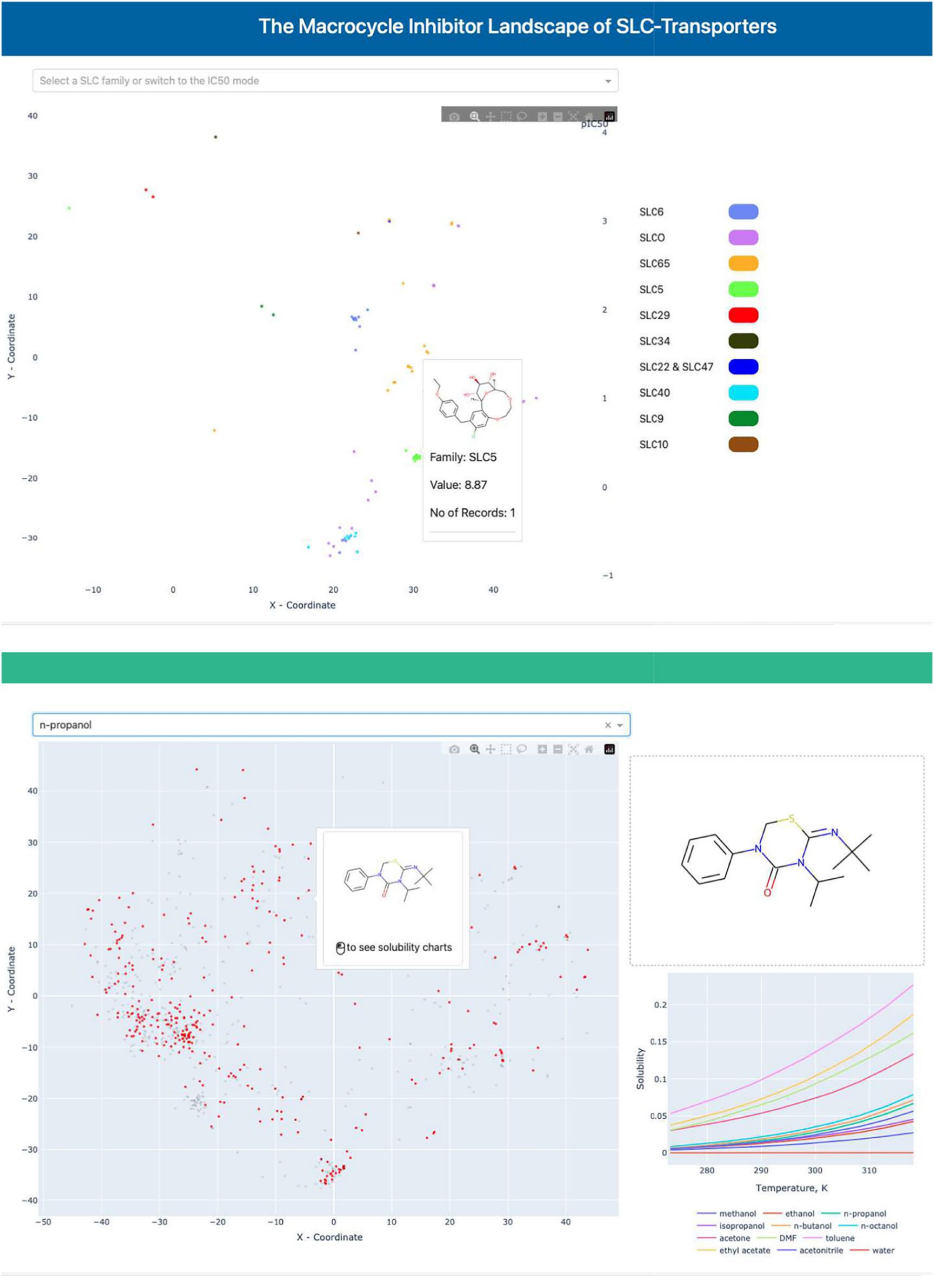
Screenshots demonstrating the customized domain-specific visualisations based on *MolCompass*:* Top:* visualisation of the macrocycle inhibitor landscape of SLC-transporters (https://macrocyc.pharmapp.univie.ac.at) *Bottom:* an interactive visualization of the chemical space of solvents and solutes represented in *BigSolDB* solubility dataset (http://bigsoldbapp.cheminfo.space)

Visualizing chemical space with respect to chemical similarity is a universal approach for studying large chemical datasets. Instead of scrutinizing the dataset row-by-row, one can plot the chemical space on a 2D surface and inspect it from a bird’s eye holistic view [4, 5]. Recently, we deployed several domain visualizations based on *MolCompass*, particularly *MolCompassViewer*: one visualizing the macrocycle inhibitor landscape of SLC-transporters [6] and another providing an interactive visualization of solvents and solutes for the *BigSolDB* solubility dataset [27]. These visualizations demonstrates how *MolCompass* can be customized for specific domains (see Fig. 6).

However, in this research, we would like to focus more on the specific applications of *MolCompass* for the visual analysis of the applicability domain. These applications include:

- Visual validation of QSAR/QSPR models
- Models’ cliffs hunting

### QSAR/QSPR models’ confidence score

QSAR/QSPR methods play an important role in medicinal chemistry, for the *in-silico* evaluation of chemical, biological and toxicological properties of drug candidates. For this purpose, the confidence of the model plays a very important role. A researcher can filter out compounds that presumably have undesired properties, based on the confidence of the model. At the same time, the scenario when the model provides an incorrect result with high confidence is heavily undesirable, and typical scaffolds, or potential regions of the chemical space where this behavior occurs should be identified and excluded from the applicability domain of the model.

We can quantify the concept of model confidence in binary classification by evaluating the probability values, which typically emerge as outcomes in most machine learning methods for binary classification. Given a probability for a compound to be active/inactive and ground truth value, one can calculate a binary cross-entropy score:

1 $$\begin{aligned} \text {Binary Cross-Entropy (Loss)} = - \left[ y \cdot \log (p) + (1 - y) \cdot \log (1 - p) \right] \end{aligned}$$

**Fig. 7:**
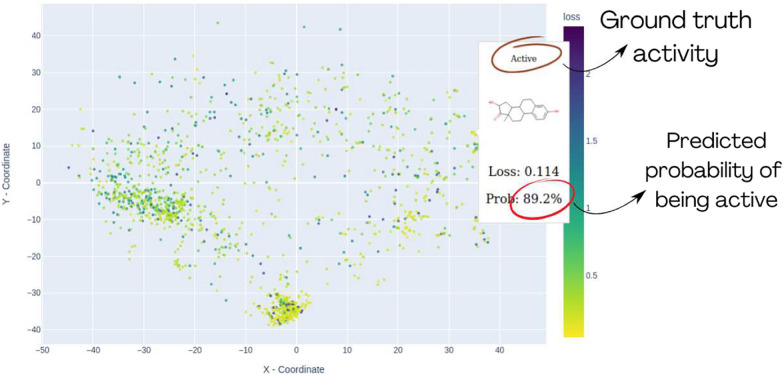
This illustrates how *MolCompassViewer* in FULL mode can be utilized for Loss analysis. Coordinates of points are determined by the *MolCompass* model, and *MolCompassViewer* runs an interactive scatter plot, with colors corresponding Loss values. By hovering the cursor over a specific molecule within the plot, users can view detailed information, including the molecular structure, ground truth activity, predicted probability and the Loss value. The visualised data is: Estrogen Binders dataset (see sec. Reference model)

where *y*—is an actual value for a molecule to be active (1) or non-active (0), and *p*—is the predicted probability of a compound to be active.

In machine learning, this score is typically used as a loss function for model training. Thus, we refer to it further as *Loss*. The higher the Loss value, the more confident the model is in its wrong prediction. *MolCompassViewer* in FULL mode can calculate and visualize Loss values for interactive analysis. The visualization of chemical space by *Loss* value provides a convenient way for the identification of problematic regions of model space and is also a key feature for hunting models’ cliffs

### Reference model

To demonstrate the method for visual QSAR/QSPR analysis, we constructed a reference QSAR model utilizing the Estrogen Binders (CERAPP) compounds dataset [28]. This dataset is binary in nature, detailing whether each compound acts as a binder to the Estrogen receptor. It encompasses 1979 compounds identified as binders and 5296 compounds recognized as non-binders.

We standardized all molecules using the *molvs* standardizer and removed counter-ions from salts. Compounds that encountered errors during the standardization process were omitted, resulting in a total of 7275 compounds remaining for analysis.

To calculate the Extended-Connectivity Fingerprints (ECFP) [29], we utilized the RDKit cheminformatics framework. For modeling, the XGboost method was used, which has demonstrated high performance in numerous QSAR/QSPR challenges [30].

The overall performance and individual probabilities were estimated using the original test set provided by the author [28].

### Visual analysis of model’s applicability domains

A standard approach to presenting the performance of QSAR/QSPR models includes providing integral statistical metrics. These metrics include: the Area Under the Receiver Operating Curve (AUC ROC), balanced accuracy, F-score, etc. However, relying exclusively on these integral values does not provide a comprehensive view of the model’s performance across different regions of the chemical space.

For an illustrative example, the reference model achieves an overall AUC ROC of 0.70, suggesting a reasonable quality level for a biological endpoint. However, a more detailed analysis conducted using *MolCompassViewer* uncovers significant shortcomings in this model.

**Fig. 8:**
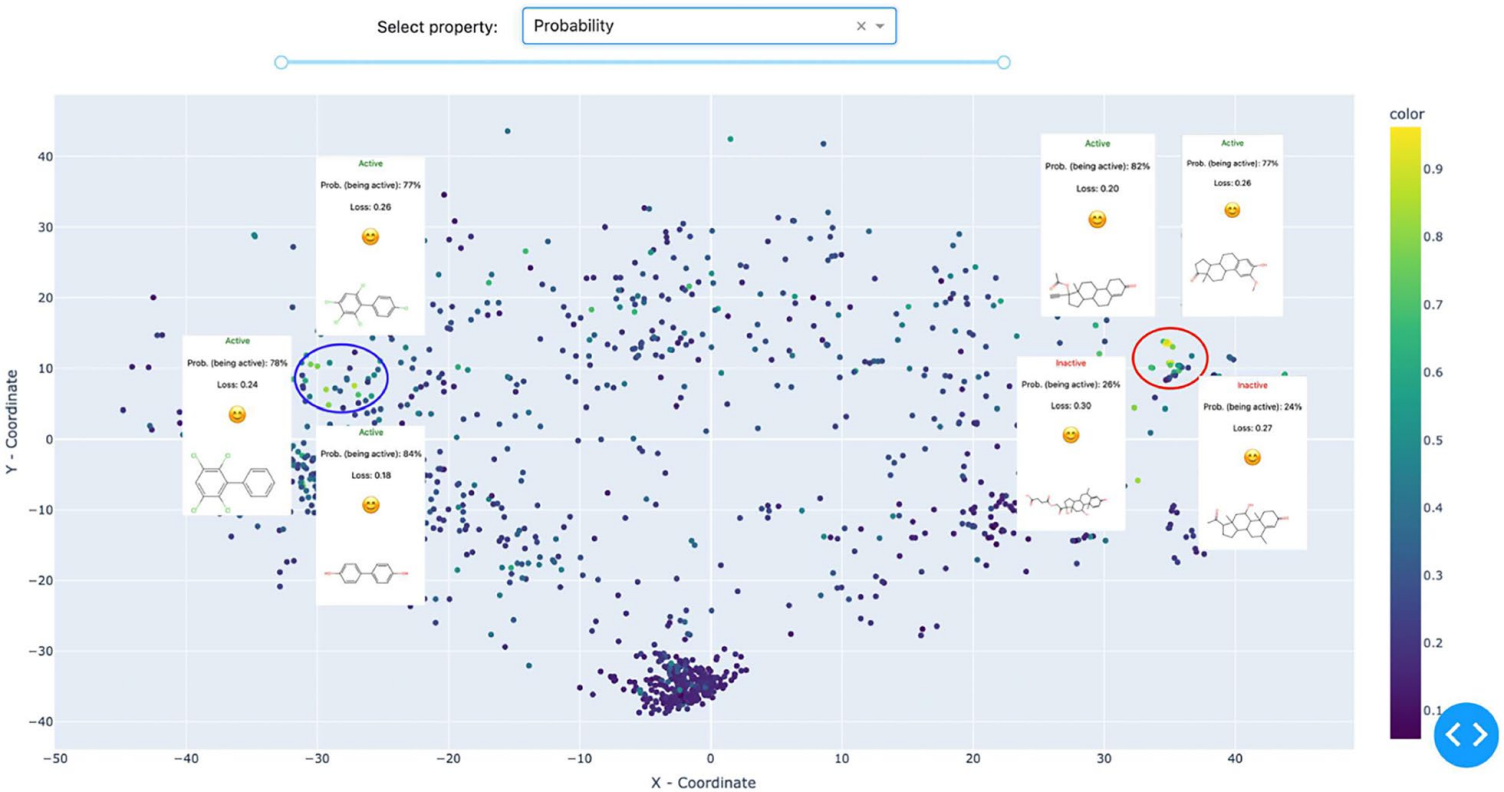
Two clusters have been attributed with high confidence by the reference model. The denser cluster on the left represents steroid derivatives, while the right, less defined cluster includes polychlorinated biphenyls and polyphenols. The visualised data is: Estrogen Binders dataset (see sec. Reference model)

In the analysis, a high-density cluster is evident at the lower part of the map, consisting solely of non-cyclic compounds (see Fig. 5 bottom). The reference model shows a higher ratio of compounds with elevated loss values in this cluster, indicating issues in model prediction. *MolCompassViewer’s* detailed analysis reveals that the majority of the predicted probabilities within this cluster are below 0.5 (top subplot on 5 bottom figure) Consequently, the model inaccurately categorizes non-cyclic compounds as non-binders. Based on visual validation, one can conclude that this area of chemical space is outside of the Applicability Domain for the model (Fig. 7).

Switching the visualization to a ’probability’ color scheme, which uses predicted probabilities for coloring, allows for identifying a dense cluster located on the right side of the chemical map (see Fig. 8). This cluster is predominantly composed of steroid derivatives. A detailed analysis reveals well-balanced predictions and robust predictive capabilities within this area, indicating that the model performs proficiently when analyzing steroid derivatives, yielding predictions with high confidence in this region of the chemical space. Such performance aligns with expectations, given that the model under investigation is tailored towards Estrogen binding. AD analysis of this area indicates that ROC AUC score for this area is 0.91, which is significantly higher than the average over the dataset (0.7).

At the same time, another cluster, albeit less distinct, is visible (Fig. 8, right cluster, encircled in blue). This cluster is enriched with polychlorinated biphenyls and polyphenols. Our literature search revealed that both families-polychlorinated biphenyls and polyphenols-are well-knows for their endocrine-disrupting activity [31, 32]. That is a good example of how the visual analysis of QSAR/QSPR models can provide some insights directly from data, which can be validated by further literature searches.

### Model cliffs hunting

“Activity cliffs” are a well-established concept that can be formulated as “the ratio of the difference in activity between two compounds to their ’distance’ of separation in a given chemical space” [33]. Building on the concept of activity cliffs, we introduce the notion of a “model cliff.” A model cliff refers to an undetected (by a model under investigation) change in binary activity between two structurally similar compounds.

**Fig. 9:**
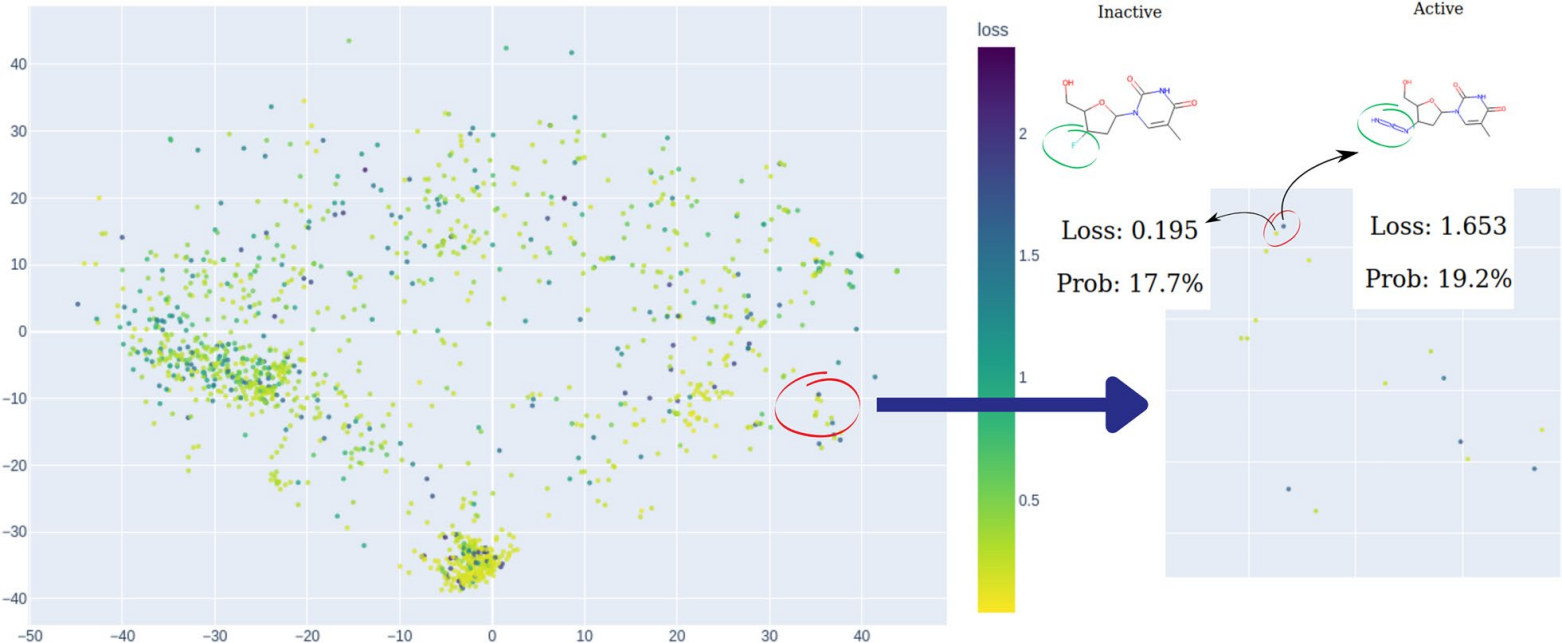
An illustration of hunting for model cliffs. On the left side of the chemical map, two points lie in close proximity yet display contrasting colors. Further investigation into this peculiar observation uncovers that although these two compounds share a high degree of structural similarity, they exhibit opposite activities, posing a challenge that the model fails to address effectively. The visualised data is: Estrogen Binders dataset (see sec. Reference model)

To identify activity cliffs, one can use “Loss” as the color scheme. In this setting, model cliffs appear as proximate points with contrasting colors. An illustrative example is presented in Fig. 9. Here, two points encircled in red share the same 1-[5-(hydroxymethyl)oxolan-2-yl]-5-methylpyrimidine-2,4-dione scaffold. However, there is a variance in their activity. The 4-fluoro derivative is inactive, consistent with the model’s prediction at an 82.3% probability of inactivity (and a 17.7% probability of activity, as seen in Fig. 9). Consequently, the loss value is low due to the correct prediction.

In contrast, the model anticipates the 4-azide derivative as inactive too, but in reality, the compound is active, resulting in a high loss value. Substituting the fluorine group with an azide at this position alters the activity, a feature that the model fails to capture. We hypothesize that this discrepancy in activities results from the potential covalent binding of the azide group to the target.

### Known limitations

It should be noted that the original parametric t-SNE model was trained on the ChEMBL dataset, so the model probably has a bias towards small organic drug-like compounds. The utilization of structural descriptors likely introduces a bias towards structural similarity between chemical compounds. Because the main goal of this study was to provide easy-to-use tools for applying the pre-trained model and discussing their applications, as well as to make the framework easier to install, use, and maintain, we decided to exclude the training code from the repository.

## Conclusions

In this research, we introduced a framework primarly designed for the Navigation in Chemical space and for the visual validation of QSAR/QSPR Models. This framework is highly flexible—it provides a python library, a KNIME node and an application for users who do not need customisation. This framework can be used for the visual analysis of chemical space, and the parts of this framework can be customized for the visualisation of specific domains. Although the framework has a wide array of possible applications, we showcased the visual validation of binary QSAR/QSPR models. The framework allows users to conduct a visual analysis of the predicted probabilities, facilitating a closer examination of the models’ confidence in these predictions. Users can identify regions in the chemical space that surpass the Applicability Domain of the model, enabling them to discern the kinds of activity changes that the model may not accurately identify. *MolCompassViewer*, enhances this process by enabling users to calculate statistical parameters for distinguished regions of chemical space individually. This provision leads to a more comprehensive understanding of the practical applicability of the QSAR/QSPR models under investigation. We believe that these tools will help with *in-deep* analysis of the models, particularly those that are used for regulatory purposes. We hope that, finally, it will pave the way for reliable and trustworthy QSAR/QSPR modeling.

## Availability and requirements

- Project name: MolCompass
- Project home page: https://github.com/sergsb/molcomplib
- Operating system(s): Platform independent
- Programming language: Python
- Other requirements: Python $\ge 3.6$, *rdkit* as a chemoinformatic engine, *molvs* for molecules standardization, *plotly* and *dash* as scientific visualisation packages
- License: MIT

## Data Availability

*molcomplib* library is freely available on GitHub https://github.com/sergsb/molcomplib. Also, it can be installed using pip (python package manager, *pip install molcomplib*). The source code of MolCompass KNIME node is also posted on GitHub https://github.com/sergsb/MolCompassKnimeNode. The recommended method for installing the MolCompass KNIME node is using pre-compiled packages available at Zenodo (https://zenodo.org/doi/10.5281/zenodo.12624632). Installation instructions and alternative methods are available on GitHub. *MolCompassViewer* is available on GitHub https://github.com/sergsb/molcompview, and can be installed using pip (*pip install molcompview*). All projects are licensed under MIT license.
